# Trends in Minimally Invasive Approaches for Liver Resections–A Systematic Review

**DOI:** 10.3390/jcm11226721

**Published:** 2022-11-14

**Authors:** Florin Graur, Razvan Alexandru Ciocan, Andra Ciocan, Ion Cosmin Puia, Emil Mois, Luminita Furcea, Florin Zaharie, Calin Popa, Diana Schlanger, Calin Vaida, Doina Pisla, Nadim Al Hajjar

**Affiliations:** 1 Department of Surgery–Surgery III, “Iuliu Hațieganu” University of Medicine and Pharmacy Cluj-Napoca, Croitorilor Street, No. 19-21, 400162 Cluj-Napoca, Romania; 2 “Prof. Dr. Octavian Fodor” Regional Institute of Gastroenterology and Hepatology Cluj-Napoca, Croitorilor Street, No. 19-21, 400162 Cluj-Napoca, Romania; 3 Department of Surgery–Practical Abilities, “Iuliu Hațieganu” University of Medicine and Pharmacy Cluj-Napoca, Marinescu Street, No. 23, 400337 Cluj-Napoca, Romania; 4 “CESTER” Research Centre for the Robot Simulation and Testing, Technical University Cluj-Napoca, Memorandumului Street, No. 28, 400114 Cluj-Napoca, Romania

**Keywords:** SILS, NOTES, laparoscopic surgery, minimally invasive, liver surgery

## Abstract

Background: SILS (single incision laparoscopic surgery) and NOTES (natural orifice transluminal endoscopic surgery) are considered breakthroughs in minimally invasive surgery, the first consisting in the surgeon working via a single entrance site and the second via a natural orifice (e.g., oral cavity). Methods: Since 2000 until 2022, the original articles published in the online databases were analyzed. Eligible studies included information about the current therapy of patients with liver surgical pathology and how the two new techniques improve the surgical approach. Results: A total of 798 studies were identified. By applying the exclusion criteria, nine studies remained to be included in the review. Two out of nine studies examined the NOTES approach in liver surgery, whereas the other seven focused on the SILS technique. The age of the patients ranged between 24 and 83 years. Liver resections for hepatocellular carcinoma or colorectal metastases were undertaken and biliary or hydatid cysts were removed. The mean procedure time was 95 to 205 min and the average diameter of the lesions was 5 cm. Conclusions: When practiced by multidisciplinary teams, transvaginal liver resection is feasible and safe. The goals of SILS and NOTES are to be less intrusive, more easily tolerated and aesthetic.

## 1. Introduction

SILS (single incision laparoscopic surgery) is considered a breakthrough in minimally invasive surgery, consisting in the surgeon working just via a single entrance site, usually the umbilicus. The practice started in the last two decades, in 2005, an acute appendicitis was treated in the department of pediatric surgery in Turkey. Since then, it has gained worldwide recognition and acceptance, and no laparoscopic surgeon today feels complete without a thorough grasp of SILS, even though it has not gained the expected popularity as when first introduced [1]. Numerous techniques exist for SILS, including: multiple face punctures via a single skin incision; extra transabdominal sutures for target organ stability; and innovative port access devices. With the added benefit of being able to switch to multiport laparoscopy, if necessary, single-incision laparoscopy claims to improve results and eliminate further incisions [2]. Better aesthetics, less blood loss, faster recovery, early return to work, versatility, more patient acceptability, and simple tissue retrieval count as the many benefits of SILS. On the other hand, the disadvantages are multiple: high costs for instruments (both trocars and hand tools); extensive expertise required; longer operating time; technically challenging maneuvers; and more difficult to manage intraoperative complications compared to laparoscopy [3].

Similar to SILS, NOTES (natural orifice transluminal endoscopic surgery) is an innovation in laparoscopic surgery, enabling incisionless endoscopic procedures via a natural orifice (oral cavity, urethra, vagina or anus). Recently, in Portugal, in addition to the single transgastric, transvaginal or transcolonic routes, a combination of transgastric and transvaginal technique for cholecystectomy has been used [4]. The most significant benefit of NOTES is its great aesthetic value, since this technique leaves no externally visible scar. Additionally, there is less need for anesthesia and analgesics, less postoperative discomfort, a quicker recovery, an early return to work and no abdominal wound problems such as seroma, hematoma or abscess. Likewise, NOTES is not devoid of its own detractors; while some of the aforementioned results hint at a hopeful future for these novel procedures, there are still substantial ethical, procedural, and technological problems that need to be answered before that promise can be realized [5]. A team of highly competent and experienced surgeons and gynecologists are needed to conduct NOTES using very complex and costly endoscopic as well as manual equipment. Flexible endoscopy is used to perform natural orifice endoscopic procedures, although most surgeons currently have little to no expertise using it in the abdominal cavity (or elsewhere). Since gastrointestinal leaks would represent a catastrophic complication that rarely occurs after routine laparoscopic cholecystectomies and appendectomies, the lack of sterilization and secure closure of the gastric or colonic wall continues to be the biggest challenge in transgastric or transcolonic NOTES. Uncertain evidence on its safety, clinical outcome, and postoperative sequelae is a further disadvantage, being still a low volume surgical procedure [5,6].

There are a variety of methods for executing SILS and NOTES. These may be used for multiple intraabdominal and pelvic surgeries, including appendectomy, gastrostomy, gastrectomy, adrenalectomy, colorectal procedures, bariatric treatments, and urological operations [7]. However, cholecystectomy remains the most frequent surgery performed with SILS and NOTES [8].

Today, SILS and NOTES used in major resections, such as liver surgery, are limited to case series publications. Therefore, the aim of our study is to draw attention to these difficult, yet resourceful surgical approaches by analyzing both their highlights and challenges.

## 2. Materials and Methods

**Figure 1:**
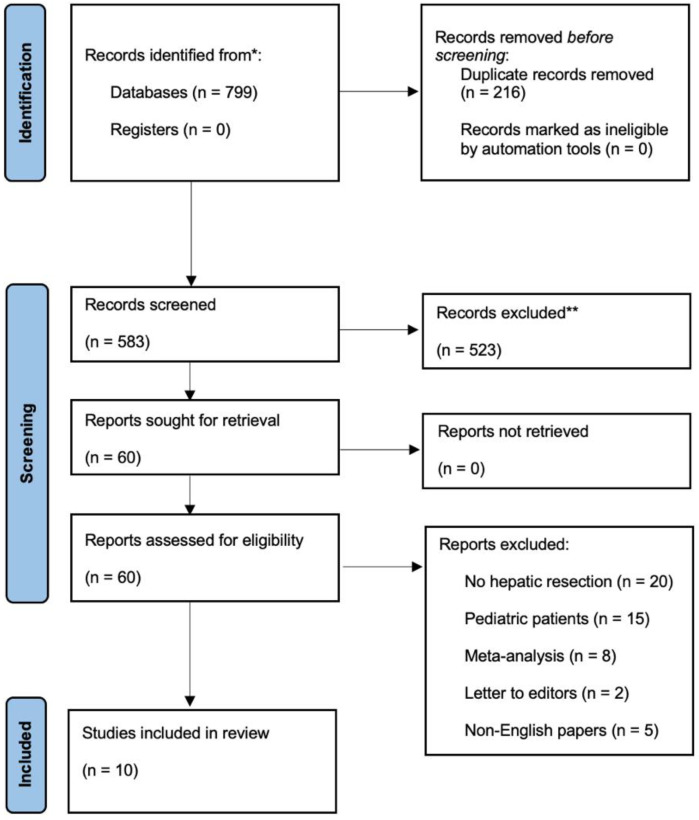
PRISMA flow chart for the selected studies included in the systematic review (* records identified from Pubmed, Embase and Cochrane database in total, ** records excluded by two different reviewers).

This systematic review’s methodology includes the definition of search techniques, selection criteria and data extraction. This research adhered to the Preferred Reporting Items for Systematic Reviews and Meta-Analyses (PRISMA) statement’s requirements (Figure 1).

From January 2000 until February 2022, the original articles published in English in the online databases Pubmed (Medline), Embase and Cochrane were analyzed. “Liver surgery” AND “NOTES” AND “SILS” AND “NOTES hepatic surgery” AND “SILS hepatic surgery” AND “Single port laparoscopy” AND “SILS hepatectomy” were used to find eligible studies. Two independent reviewers conducted the screening, based on a preset of inclusion criteria, in order to expend research and reduce bias.

Selection standards: eligible studies included information about the current therapy of patients with liver surgical pathology and how the two new techniques improve the surgical approach; type of study: original article; type of participation: cohort age and gender, diagnosis; type of intervention: NOTES or SILS with/without need for conversion to laparoscopy or open; type of outcome measures: pathology addressed, liver tumors; type of liver resection, procedure time in minutes, intraoperative blood loss in milliliters, recurrence rate, intra- or postoperative complications.

Exclusion criteria: letters to editor, short reports, meta-analyses, non-English papers, non-surgical patients, no hepatic resection, palliative care alone, patients under 18 years old.

We used an Excel extraction form for data collection. For the quality assessment we utilized study participation, factor measurement, relevance and applicability. After a comprehensive examination of all qualifying papers, two independent reviewers extracted data and double-checked all results. During the selection and extraction of data, any discrepancies between the two reviewers were reviewed with a third and fourth reviewer. The reference lists of certain research were combed for prospective papers using the “snowball” technique.

To meet the objectives of our study, we analyzed the original articles by comparing: the total number of patients operated on with a certain technique (NOTES or SILS); whether conversion to open or laparoscopic approach was necessary; the type of liver lesions resected (malignant or benign: hepatocellular carcinoma, adenoma, biliary cysts, hydatid cysts etc.); intraoperative characteristics (tumor location, diameter of the liver lesion, mean procedural time, blood loss); the way of approach, intraoperative and postoperative complications; and if postoperative recurrence was identified. To express the degree of consistency in the examined article’s results, we have displayed tables for quantitative data (extreme values of the cohort and mean value calculated) and qualitative data (SILS/NOTES, gender, pathology, technique, recurrence etc.) with a textual description and content analysis for in depth evaluation. For a standardized presentation, we settled centimeters, minutes and milliliters as standard units of measurement, the numbers were converted from millimeters, hours or liters, whenever necessary. For the missing data, we wrote “N.A.–not available”, and it was not taken into consideration for calculating the variable’s mean, median or other derivatives.

## 3. Results

By scanning Pubmed/Medline, Embase and Cochrane databases, a total of 798 studies were identified. After eliminating duplicates, 582 titles and abstracts were reviewed. After screening, 523 papers were removed, leaving 59 publications, which were evaluated in depth. By applying the exclusion criteria, ten studies remained to be included in the review (Figure 1), published from 2008 until 2016.

Two out of nine articles included in the study examined the NOTES technique in liver surgery, whereas the other seven focused on the SILS approach (Table 1). The majority of studies were case reports. Two studies considered minor liver resections, three studies considered the excision of liver cysts, one study highlighted the drainage of a liver hematoma, two studies focused on segmental liver resection, and lastly, one study summarized 17 patients, who underwent liver surgery for various types of liver lesions using the SILS technique.

Regarding the NOTES technique, the average age of the patients analyzed in the two studies was 61.5 years (Table 2). Both of the NOTES techniques required laparoscopic assistance. One approach was used in a series of cases for liver cysts removal and the second presented a unique metastasis localized in segment V of the liver according to the Couinaud classification, with the primary lesion of colorectal cancer, previously treated with systemic oncological therapy.

The approach was different between the two studies. In the first one, a transvaginal approach was used, the intervention duration was 95 min; while in the second study, a gastric approach was chosen with an average operating time of 101 min. The lesions’ sizes varied from 3 to 10 cm in diameter, with a mean width of 8 cm. In both studies, there were no complications and no re-interventions required. In one patient of Wang’s study, laparoscopic assistance was required due to the inadequate exposure of the cyst, because of the posterior location, in segment VIII (Table 3). A total of 58 patients, aged between 24 and 83 years have undergone liver surgery through the SILS approach with a mean age of 50, out of which four needed conversion to open surgery. There were more male patients in the cohort examined; M:F = 28:23. The type of resection varied upon the type of liver lesion, implying 35 minor liver resections–segmentectomy or bisegmentectomy for hepatocellular adenomas or carcinomas, 12 metastasectomies, two liver hemangioma resection, one focal nodular hyperplasia resection, five biliary cysts removal and four hydatid cysts removal. The lesions resected were easily accessible, superficially located in segments II, III, V and VI. Regarding the perioperative complications, massive intraoperative bleeding was the cause for conversion and one case of SILS cholecystectomy resulted in a large subcapsular hematoma as an immediate postoperative complication, with severe anemia and repeated packed red blood cells transfusions needed, which was solved by a SILS reintervention with hematoma drainage and hemostasis. There was no need for conversion to laparoscopy or open surgery and the evolution was favorable. Considering the primary malignant tumors, four cases presented the recurrence of hepatocellular carcinoma during the oncological follow-up (Table 4 and Table 5). In total, there were four conversions to open surgery, none to multiport laparoscopy and one required laparoscopic assistance during the main procedure.

The mean tumor size in the SILS treated patients was 5 cm wide, ranging from 1 to 14 cm in diameter. The liver was approached via the umbilicus through a single-port, excluding the cases that needed conversion to open surgery and only one case presented an infraumbilical incision. The supine position, in Anti-Trendelenburg with the legs apart in order to facilitate the movements of the primary surgeon was favored in all cases, where the technique was thoroughly described. The median hospital stay was 5 days, ranging from 3 to 13 days. The interventions lasted between 55 and 545 min, with an average procedure time of 156 min. The conversion rate to multiport laparoscopy was 0% and to open surgery was 7%. The intraoperative blood loss was less than 50 mL in two cases evaluated and more than 2000 mL in others, the mean amount was 290 mL, with necessary packed red blood cells transfusions accordingly. Hansen et al. had missing data regarding tumor size and procedure duration. Similarly, Erylimaz’s study did not provide evidence regarding procedure time and the amount of intraoperative blood loss, increasing the risk of bias.

## 4. Discussion

In the last decades, laparoscopic liver resection has undergone remarkable expansion. Even malignant tumors have been treated laparoscopically, with evidenced based benefits in terms of survival and decreased invasiveness. The minimally invasive liver surgery may be even further evaluated when two other novel approaches are considered [19].

The first, SILS, is a pure laparoscopic procedure, whereas the second is a hybrid laparoscopic procedure. NOTES liver resection has the objective of even less invasiveness, approaching the intraperitoneal organs through hollow viscus located in the proximity of the targeted lesion. This procedure is practiced by competent teams, with extensive expertise in minimally invasive surgery, since it may result in postoperative serious complications, like visceral damage or fistula, with consecutive peritonitis. In fact, to avoid peritonitis in some sectors, authors paired hybrid NOTES with extra minilaparoscopy. As an alternative, SILS liver resection has been employed. Because SILS is constrained by the co-axial arrangement of its tools, some surgeons undertake the surgical operation using the cross-hand approach. However, these issues may be resolved by inserting the bilateral laparoscopic ports as profoundly as feasible. Considering other surgeons’ experience, the cross-hand approach is not always required, for example in SILS cholecystectomy. Endoscopic forceps are not fit for combat. The up-to-date literature hypothesizes that SILS liver resection may be achieved utilizing the parallel method, albeit this is dependent on the tumors’ location [20,21,22].

SILS blends the aesthetic benefit of NOTES with the technical familiarity of the typical multiport laparoscopic technique. In this publication, the authors describe a unique approach of SILS liver resection for hepatocellular carcinoma in a patient with compensated liver cirrhosis. Comparing the usual multiport strategy to performing laparoscopic surgeries, there may be advantages to using fewer ports. These include fewer scars, reduced need for narcotic analgesics, earlier hospital discharge, faster healing, and less pain in the abdominal wall [3].

The closure of the natural orifices, via which the abdominal cavity is accessed, is one of the most important safety elements of NOTES. Despite the description of several systems, there are no confirmed procedures in terms of safety and effectiveness for stomach or bowel orifices closure. Before these devices can be passed via an endoscopic working channel and utilized to suture securely, conveniently, and rapidly, they must be made less cumbersome and undergo more refinement. In contrast, the vagina allows simple, secure access, and colpotomy is a safe gesture that gynecologists have frequently performed for decades. Occasionally, the transvaginal approach has been recorded for the removal of non-gynecological organs, such as nephrectomy and hemicolectomy specimens. Except for modest vaginal soreness in the first week after surgery, posterior colpotomy is essentially painless. Infrequent complications of colpotomy include urinary bladder lesions, pelvic hematomas, urinary tract infection, and surgical site infection. Dyspareunia can be a potential consequence after posterior colpotomy, however the probability of developing clinically significant dyspareunia is a rarity. The extent of the hemorrhage and the fast resolution of the liver function may have a significant impact on the length of the postoperative hospital stay. However, for young female patients with benign tumors, a single incision offers better cosmetic outcomes [23,24,25].

Because of the restricted operational field and two-instrument limitation, SILS is technically more difficult than traditional laparoscopic surgery. The resection of deep-seated lesions, for which the vision is obscured, is particularly difficult and dangerous. Nevertheless, hemorrhage may be efficiently managed with the use of tourniquets, bipolar electrocautery, ultrasonic scalpels, absorbable clips, vascular staplers, ligatures and biological glues. Large arteries and, more importantly, bile ducts should be divided using staplers and then sutured with absorbable ligature as necessary, in order to decrease the risk of postoperative bilioma formation [26].

The advantages of SILS include a hidden incision for better cosmetic results, the minimization of secondary abdominal injury, milder postoperative pain, quicker recovery, earlier return to daily activities, and shortened hospital stay, which all comprise a positive impact on patient’s rehabilitation [27]. When talking about the disadvantages, the costs represent the major burden; sophisticated instrument expenses, and the cost of the formation of a highly skilled multidisciplinary team. Moreover, when there is a need for specimen extraction in one piece, the incision’s length in not always enough and the need for its extension makes it harder for the patient to accept the procedure with all its risks, compared to a normal laparoscopic approach. Another drawback is the duration of the procedure, which is very long and sometimes the surgeon feels the urge of conversion to conventional laparoscopy, and, more importantly, when there is a difficult dissection and complications appear, such as important hemorrhage or bile leak [28].

Similarly, in NOTES, explaining the safety and complication rate of the procedure to the patient is difficult. It comes with no external scar or parietal injury, nor surgical site infection or incisional hernia, but there is an extremely high cost of the procedure, and the need of an experienced team. The single port approach has a steep learning curve and is demanding. In contrast to multi-port surgery and open surgery, this approach requires surgeons to overcome in-line vision and the absence of operative triangulation, referred to as the “chopstick effect” [29]. In NOTES, all members of the surgical team should have high quality skills, both the surgeon and gynecologist, when the vaginal orifice is used, and it cannot be conducted with the help of inexperienced assistants, as in conventional laparoscopy. Additionally, the conversion to laparoscopy whenever it is needed is more difficult than in SILS, especially in liver surgery, due to the organ’s propensity to bleed and when access to the hepatic veins or inferior vena cava is difficult to obtain.

While SILS and NOTES are important breakthroughs in minimally invasive surgery, and when Escobar et al. first reported a robotic SILS gynecologic procedure using the da Vinci S robot and GelSeal cap in 2009, proving robotic-assisted single-port surgery feasible, it paved the road to application of this method in liver surgery [30]. The endeavor to expand the indications for robotic surgery in conjunction with NOTES or SILS opens the door for less complications, consecutively both surgeons and patients being more open to accept these approaches in liver surgery. With improved innovations in instruments, cameras, and scopes, as well as the refinement of surgical techniques, the difficulties associated with performing SILS have been gradually diminished, but not at all eliminated over time. With the development of new port designs, pre-bent tools, retraction devices, the use of intra-abdominal sutures for traction, unique camera holding systems, and revolutionary surgical procedures, the practicality of routinely performing SILS has risen, and NOTES has been made possible. Surgical techniques are increasingly customized not just to the patient’s expectations about surgical results and surgical efficiency, but also to the patient’s basic pathology.

The limitations of our systematic review emerge from the lack of standardized maneuvers and unusual situations included in our study. The majority of studies were case reports, here deriving the main source of bias, the heterogeneity of the data presentation, and not all denominating each variable we took into consideration. The diminished level of knowledge on the topic addressed is implied by the scant number of papers present in the literature. Therefore, it is impossibile to apply an extremely strict exclusion criteria, the data being heterogeneous and based on the number of cases treated in each article, the mean operative time, the volume of blood lost, and complications—barriers that heavily depend on the preferences or resources of the researcher. To assess the safety, effectiveness, complication rates, and possible benefits, if any, that these cutting-edge procedures may offer, randomized studies contrasting single-incision laparoscopy and natural orifice endoscopic surgery, with conventional laparoscopy, are required.

## 5. Conclusions

In conclusion, the single-incision transumbilical approach predicts the future path of minimally invasive surgery, including liver resections. As time passes, more effective devices are expected to be developed in order to keep up with the surgeon’s need, decrease intraoperative complications and expand indications. Moreover, the advantages of SILS liver resections must be examined in larger series of cases and, if feasible, a prospective randomized trial. When practiced by multidisciplinary teams, transvaginal liver resection is feasible and safe. The goal of natural orifice endoscopic surgery is to be less intrusive, more easily tolerated and aesthetic. It will likely pave the way for not only significant medical, but also technical, advancements.

## Figures and Tables

**Table 1:** General overview of the included studies.

Author	Year	Technique	Number of Patients	Objectives
Noguera, J.F. et al. [9]	2008	NOTES	1	Liver resection
Shetty, G.S. et al. [10]	2012	SILS	24	Liver resection
Hansen, A.J. et al. [11]	2011	SILS	1	Liver hematoma drainage
Kashiwagi, H. et al. [12]	2011	SILS	1	Biliary cyst removal
Dapri G. et al. [13]	2012	SILS	6	Focal hepatic lesions resection
Wu, S. et al. [14]	2014	SILS	17	Focal hepatic lesions resection
Eryilmaz, R. et al. [15]	2014	SILS	1	Hydatid cyst
Claude, T. et al. [16]	2014	SILS	7	Focal hepatic lesions resection
Igami, T. et al. [17]	2015	SILS	1	Biliary cyst removal
Wang, D. et al. [18]	2016	NOTES	4	Biliary cyst removal

**Table 2:** General overview of NOTES patients.

Author	Sex	Age (Years)	Pathology	Technique
Noguera, J.F. et al. [9]	F	61	Colorectal liver metastasis	NOTES combined with laparoscopy
Wang, D. et al. [18]	M–3 F–1	38–82	Liver cysts	NOTES combined with laparoscopy

**Table 3:** NOTES: analysis of intraoperative characteristics, approach and postoperative recurrence.

Author	Tumor Size (Diameter in Centimeters)	Way of Approach	Mean Procedure Time (min)	Recurrence
Noguera, J.F. et al. [9]	3	Transvaginal	95	No
Wang, D. et al. [18]	10	Transgastric	101	No

**Table 4:** General overview of SILS patients.

Author	Sex	Age (Years)	Pathology	Technique
Shetty, G.S. et al. [10]	M–20 F–4	34–82	Hepatocellular carcinoma	Single Incision Laparoscopic Open conversion
Hansen, A.J. et al. [11]	F	25	Liver Hematoma	Single Incision Laparoscopic
Kashiwagi, H. et al. [12]	F	83	Liver cyst	Single Incision Laparoscopic
Dapri, G. et al. [13]	F–6	24–65	Biliary cyst Hydatid cyst Colorectal liver metastases	Single Incision Laparoscopic
Wu, S. et al. [14]	M–8 F–9	31–71	Liver hemangioma Hepatocellular adenomas Hepatocellular carcinomas Colorectal liver metastases	Single Incision Laparoscopic
Eryilmaz, R. et al. [15]	F	17	Hydatid cyst	2 cm Infraumbilical incision
Igami, T. et al. [17]	F	80	Huge Liver Cyst	Z Shaped Umbilical Incision
Claude, T. et al. [16]	M–3 F–4	31–71	Focal nodular hyperplasia Colorectal liver metastases Hepatocellular carcinoma Hepatocellular adenoma	Single Incision Laparoscopic

**Table 5:** SILS: analysis of intraoperative characteristics, approach and postoperative recurrence.

Author	Mean Tumor Size (Diameter in Centimeters)	Way of Approach	Mean Procedure Time (min)	Recurrence	Average Blood Loss (mL)
Shetty, G.S. et al. [10]	3.6 (1–9)	Umbilical	205 (95–545)	4	500 (100–2500)
Hansen, A.J. et al. [11]	N.A.	Umbilical	N.A.	No	Minimum
Kashiwagi, H. et al. [12]	>10	Umbilical	130	No	5
Dapri, G. et al. [13]	N.A.	Umbilical	126 (89–125)	No	275 (40–500)
Wu, S. et al. [14]	1.5–10.5	Umbilical	55–185	No	30–830
Eryilmaz, R. et al. [15]	8.9	Infraumbilical	N.A.	No	N.A.
Igami, T. et al. [17]	14	Umbilical	169	No	51
Claude, T. et al. [16]	2.4 (2–4.7)	Umbilical	110 (60–150)	N.A. *	50 (25–150)

* N.A.—not available.

## Data Availability

Not applicable.
